# Clinical Symptoms Influencing Parkinson’s Patients’ Quality of Life in Latvia: A Single-Center Cohort Study

**DOI:** 10.3390/medicina59050935

**Published:** 2023-05-12

**Authors:** Olga Minibajeva, Estere Zeltiņa, Guntis Karelis, Nataļja Kurjāne, Viktorija Ķēniņa

**Affiliations:** 1 Department of Doctoral Studies, Rīga Stradiņš University, LV-1007 Rīga, Latvia; 2 Department of Neurology and Neurosurgery, Riga East University Hospital, LV-1079 Rīga, Latvia; guntis.karelis@gmail.com; 3 Faculty of Medicine, Rīga Stradiņš University, LV-1007 Rīga, Latvia; 033798@rsu.edu.lv; 4 Department of Infectology, Rīga Stradiņš University, LV-1006 Rīga, Latvia; 5 Department of Biology and Microbiology, Rīga Stradiņš University, LV-1007 Rīga, Latvia; natalja.kurjane@rsu.lv (N.K.); kenina.viktorija@gmail.com (V.Ķ.)

**Keywords:** Parkinson’s disease, clinical phenotypes, non-motor symptoms, quality of life

## Abstract

*Background and Objectives:* Parkinson’s disease (PD) is a chronic, progressive illness with a profound impact on health-related quality of life, and it is crucial to know what factors influence the quality of life throughout the course of the disease. This study aimed to evaluate PD patients’ motor and non-motor symptoms to compare symptom severity between PD clinical phenotypes and to assess the impact of disease symptoms on quality of life in a cohort of Latvian patients. *Materials and Methods:* We evaluated 43 patients with Parkinson’s disease. Fourteen patients had tremor dominant (TD) PD, twenty-five patients had postural instability/gait difficulty (PIGD), and four patients had a mixed phenotype. *Results:* The patients’ mean age was 65.21 years, and the disease’s mean duration was 7 years. The most common non-motor symptoms were fatigue (95.3%), sleep disturbance (83.7%), daytime sleepiness (83.7%), and pain and other sensations (81.4%). PIGD patients had a higher prevalence of depressed mood, daytime sleepiness, constipation, lightheadedness on standing, cognitive impairment, and severe gastrointestinal and urinary disturbances (as assessed using the SCOPA-AUT domains) compared with TD patients. A high prevalence of fatigue was assessed in both disease subtypes. Health-related quality of life significantly statistically correlated with MDS-UPDRS parts III and IV (r = 0.704), the Hoehn and Yahr scale (r = 0.723), as well as the SCOPA-AUT scale’s gastrointestinal (r = 0.639), cardiovascular (r = 0.586), thermoregulatory (r = 0.566) and pupillomotor domains (r = 0.597). *Conclusions:* The severity of motor symptoms, as well as non-motor symptoms, such as fatigue, apathy, sleep problems and daytime sleepiness, pain, and disturbances in gastrointestinal and cardiovascular function, negatively affect PD patients’ health-related quality of life. Thermoregulatory and pupillomotor symptoms also significantly affect PD patients’ well-being.

## 1. Introduction

Parkinson’s disease (PD) is a neurodegenerative disease characterized by the loss of dopaminergic neurons in the substantia nigra pars compacta in the midbrain [1]. The histopathological hallmark of the disease is Lewi bodies that are intracellular aggregates, with alpha-synuclein being the major pathognomonic component [2].

PD commonly affects people older than 65 years; 90% of PD cases are sporadic. In 10% of PD cases, familial forms of gene abnormalities can cause the disease, including mutations in the SNCA, LRRK2, and PARK2 genes [3].

PD affects 1–2 people per 1000, and 1% of people older than 60 years [4]; with an ageing population, this number is expected to increase. A systematic analysis in the Global Burden of Disease Study 2016 described the increasing number of PD cases, making it one of the most worrying neurodegenerative diseases. In 1990, 2.5 million people were diagnosed with PD worldwide, whereas in 2016, 6.1 million people were diagnosed with PD. It was calculated that PD caused 3.2 million disability-adjusted life years in 2016 (the number of years lost due to the disease) [5]. These data show how significant an impact PD has on patients’ lives.

PD diagnosis is mainly based on typical clinical symptoms—resting tremor, rigidity, and bradykinesia [6]. However, it has been proven that there are symptoms that develop 12–14 years before specific motor symptoms appear; collectively, these are called prodromal Parkinson’s disease [7]. The most common non-motor PD manifestations are sleep disturbances, autonomic dysfunction (constipation, daytime urinary urgency, and symptomatic orthostasis), hyposmia, and psychiatric symptoms, such as depression, anxiety, or hallucinations [8].

Bradykinesia is described as slowness of movement and is caused by basal ganglia dysfunction. It also causes difficulties in initiating action activities [9]. Rigidity is defined as increased resistance and “cogwheel” phenomenon; resting tremor is characterized as a tremor with a 4–6 Hz frequency, which frequently affects the hands [10].

All these symptoms can make a person’s life disabling. A 2021 systematic review by Na Zhao et al. concluded that PD patients have a lower quality of life (QoL) in most domains compared to healthy individuals, especially regarding physical function and mental health [11].

Our study aimed to evaluate the frequency and severity of non-motor symptoms in PD patients, compare the severity of non-motor symptoms and motor scores between PD subgroups, and evaluate the impact of disease symptoms on patients’ quality of life.

## 2. Materials and Methods

**Figure 1:**
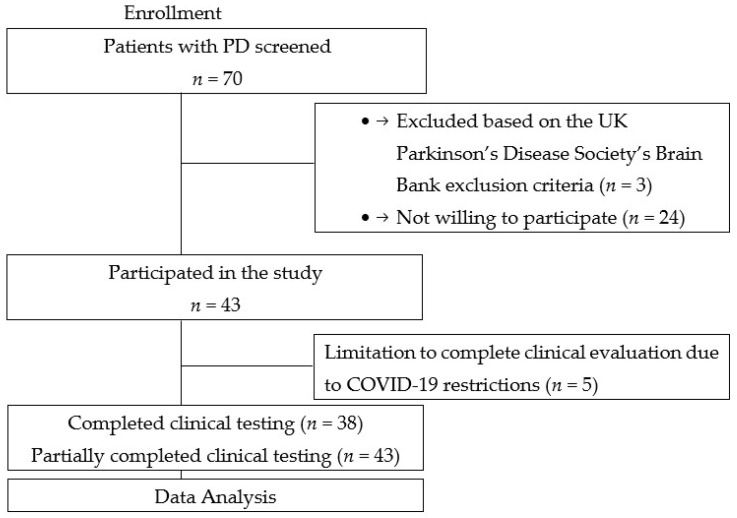
Flowchart of this study.

A total of 43 PD patients were consecutively recruited from the Outpatient Department and the Department of Neurology and Neurosurgery, Riga East University Hospital Clinic Gaiļezers, from 2019 to 2020 (Figure 1). Patients were diagnosed with Parkinson’s disease according to the UK Parkinson’s Disease Society’s Brain Bank criteria [12].

Demographic variables, including gender, age, age of onset, disease duration, and clinical type, were recorded for all participants. According to the method for clinical phenotypes classification, participants were divided into tremor dominant (TD), postural instability/gait difficulty (PIGD) and mixed phenotypes of Parkinson’s disease [13]. A professionally trained and qualified neurologist evaluated patients.

PD patients were evaluated using the Movement Disorder Society-sponsored revision of the Unified Parkinson’s Disease Rating Scale (MDS-UPDRS) during ON condition. The MDS-UPDRS has four parts: I: non-motor experiences of daily living; II: motor experiences of daily living; III: motor examination; and IV: motor complications. Each patient answered 20 questions. Item-specific instructions were provided [14]. Disease severity was evaluated using the Hoehn and Yahr scale (staging 1–5) [14,15].

Cognitive impairment was screened using the Montreal cognitive assessment (MoCA) test [16]. All dysautonomic symptoms were assessed using the self-reported scale for outcomes in PD for autonomic symptoms (SCOPA-AUT). The SCOPA-AUT is composed of six domains: gastrointestinal function, urinary function, cardiovascular function, thermoregulatory function, pupillomotor function, and sexual function [17].

PD patients’ health-related quality of life was assessed using the 39-item Parkinson’s Disease Quality of Life Questionnaire (PDQ-39) [18].

The Science Department approved the study protocols of the Riga East University Hospital Clinic Gaiļezers and the Central Medical Ethics Committee of Latvia. All participants provided written informed consent. This study adhered to the Declaration of Helsinki regarding ethical principles for medical research involving human subjects (2008).

The Movement Disorders Society-sponsored revision of the Unified Parkinson’s Disease Rating Scale, the 39-item Parkinson’s Disease Quality of Life Questionnaire and the self-reported scale for outcomes in PD for autonomic symptoms were adapted to the Latvian language, ensuring the translation from English to Latvian and the re-translation of the Latvian text into English by two independent specialists.

Statistical analyses were performed using SPSS Statistics 26.0 (Chicago, IL, USA). Data were not normally distributed and non-parametric tests of significance were used. To compare continuous data we used the Mann–Whitney test (independent samples), Fisher’s exact test, and the Chi-square test. Spearman’s rank correlation coefficients were calculated to determine the strength and direction of associations between variables. The statistical significance of the results was accepted at *p* < 0.05 (2-tailed).

## 3. Results

There were 43 patients included in the research, 23 females (53.5%) and 20 males (46.5%). The patients’ mean age was 65.21 (SD 8.935) years, and the mean duration of the disease was 7 (min 4; max 11) years. Distributing patients based on their Hoehn and Yahr (HY) stages, 17 patients were included in HY stage 1 (39.5%), 15 in HY stage 2 (34.9%), and 11 in HY stage 3 (25.6%).

According to the clinical phenotypes, participants were divided into tremor dominant (TD) (*n* = 14), postural instability/gait difficulty (PIGD) (*n* = 25), and mixed (*n* = 4) phenotypes. As the mixed phenotype group contained only four people, it was not used in the comparisons.

All 43 patients had at least one non-motor symptom. The most frequently noted complaints were related to fatigue, followed by sleep problems and daytime sleepiness, pain and other sensations, urinary and constipation problems, and depressed mood. There were statistically significant differences between PD phenotypes. Constipation problems, daytime sleepiness, lightheadedness on standing, and depressed mood were more frequently observed in PD patients with the PIGD phenotype. There were no significant differences concerning fatigue, cognitive impairment, anxious mood, apathy, sleep and urinary problems, pain and other sensations between the groups. The frequency of non-motor symptoms, according to MDS-UPDRS part I, and a comparison of these symptoms between TD and PIGD phenotypes, are presented in Table 1.

The self-reported SCOPA-AUT questionnaire was completed by 42 PD patients. Only 20 of 42 patients completed the SCOPA-AUT sexual function section. Dysautonomic symptoms were measured in all six domains. The most frequent changes were observed in the urinary and gastrointestinal domains, followed by cardiovascular and thermoregulatory function. Regarding SCOPA-AUT scores, there were observed differences between Parkinson’s disease subgroups. Patients with PIGD had severe gastrointestinal and urinary disturbances and higher SCOPA-AUT total scores compared with the TD phenotype. No statistically significant differences were obtained in the SCOPA-AUT’s cardiovascular, thermoregulatory, pupillomotor, or sexual function sections. 

Thirty-eight PD patients were screened for cognitive impairment using the MoCA test. Seventeen patients (44.7%) had mild cognitive impairments, one patient (2.6%) had moderate cognitive problems, and twenty patients (52.6%) had normal cognitive function. There were differences between the PD subgroups; in the TD group, cognitive impairments were found in two patients (18.2%), and in the PIGD group in fifteen patients (65.2%), *p* = 0.036.

All PD patients’ motor symptoms were assessed according to the MDS-UPDRS scale, parts I–IV. In the study patient group, 17 patients (39.5%) had no motor fluctuations, and 26 patients (60.5%) had a wearing-off phenomenon or dyskinesia, or both. In the TD phenotype group, motor fluctuations were present in 3 patients (21.4%), whereas in the PIGD group, they were observed in 21 patients (84%).

There were also statistically significant differences in PD motor symptoms assessments between groups. Patients with PIGD had a higher evaluation on the Hoehn and Yahr scale, all parts of the MDS-UPDRS, and total score. Non-motor and motor score differences among Parkinson’s disease phenotypes are presented in Table 2.

Health-related quality of life for 42 PD patients was assessed according to the PDQ-39. The median PDQ-39 score was 34.50 (14.75; 60.50). Subgroup analyses showed statistically significant differences (*p* = 0.001) between the PIGD 46.00 [24.50–78.50] and TD 18.00 [10.00–38.50] phenotypes. Patients with the PIGD phenotype had severe Parkinson’s disease symptoms presentation and worse health-related quality of life.

Spearman’s correlation coefficient was calculated between the PDQ-39 index and different clinical parameters and scales. There was a moderate and statistically significant (r = 0.529, *p* < 0.001) correlation between PDQ-39 results and the duration of the disease. Patients with longer disease duration had higher PDQ-39 questionnaire scores and worse quality of life.

The PDQ-39 index correlation was moderately positive and statistically significant with cognitive impairment (r = 0.571, *p* < 0.001), apathy (r = 0.600, *p* < 0.001), sleep problems (r = 0.581, *p* < 0.001), daytime sleepiness (r = 0.596, *p* < 0.001), pain and other sensations (r = 0.640, *p* < 0.001), lightheadedness on standing (r = 0.663, *p* < 0.001), and fatigue (r = 0.583, *p* < 0.001). In comparison, correlations with depression, anxiety, and constipation were mildly positive and statistically significant. However, PDQ-39 correlations with urinary problems and hallucinations and psychosis were not statistically significant. Spearman’s correlation was not calculated in the groups with DDS characteristics, as there were not enough people in the subgroups to perform this correlation.

The PDQ-39 index correlation was mild and statistically significant (r = 0.438, *p* = 0.006) with cognitive impairment as measured using the MoCA test.

Health-related quality of life, measured using the PDQ-39, correlated significantly and positively (r = 0.654, *p* < 0.001) with the total SCOPA-AUT index. Significant and moderate positive correlations were also obtained with gastrointestinal (r = 0.639, *p* < 0.001), cardiovascular (r = 0.586, *p* < 0.001), thermoregulatory (r = 0.566, *p* < 0.001), and pupillomotor domains (r = 0.597, *p* < 0.001). Correlations with the SCOPA-AUT’s urinary function and sexual function scores were not significant.

The PDQ-39 index correlation was highly positive and statistically significant with MDS-UPDRS part I (r = 0.778, *p* < 0.001), MDS-UPDRS part II (r = 0.784, *p* < 0.001), MDS-UPDRS parts III and IV (r = 0.704, *p* < 0.001), and the MDS-UPDRS total score (r = 0.837, *p* < 0.001). The correlation between the PDQ-39 and the Hoehn and Yahr scale was also highly positive and statistically significant (r = 0.723, *p* < 0.001). All obtained correlations are described in Table 3.

## 4. Discussion

In recent years, PD patients’ quality of life and the factors affecting it have been studied. The aim of our study was to assess motor and non-motor symptoms and their impact on PD patients’ well-being. PD patients’ quality of life depends on the severity of motor and non-motor symptoms. Recently, it has been indicated that autonomic dysfunction should be viewed as an important PD characterization [19].

In our study, all patients had at least one non-motor symptom; the most common non-motor symptoms were fatigue (95.3%), sleep disturbance (83.7%), daytime sleepiness (83.7%), and pain and other sensations (81.4%). Urinary problems were present in 69.8% of cases, constipation in 65.1% of cases, depressed mood in 64.3% of cases, lightheadedness on standing in 58.1% of cases, apathy in 57.1% of cases, anxious mood in 42.9% of cases, features of dopamine dysregulation syndrome in 14.3% of cases, and hallucinations and psychosis in 7.1% of cases.

Similarly, previous studies have documented a frequency of depression in up to 70% of cases [20], anxiety in up to 60% of cases [21], sleep problems in up to 92% of cases [22], pain in up to 82% of cases [23], hallucinations in up to 9.9% of cases [21], and psychosis in up to 26% of cases [24].

Previous studies documented slightly lower prevalences of orthostatic hypotension symptoms in up to 53% of cases [21], of daytime sleepiness in 20 to 76% of cases [25,26], of constipation in up to 61% of cases [27], and of DDS features in up to 8.8% of cases [28].

Our study showed a higher frequency of urinary problems, apathy, and fatigue compared to previous studies, in which urinary problems were observed in 54% of cases [21], apathy in up to 48% of cases [20], and fatigue in up to 81% of cases [29].

There was a higher frequency of cognitive impairment according to MDS-UPDRS part I results. However, an assessment of cognitive function according to the MoCA test showed mild cognitive impairment in 44.7% of cases and moderate impairment in 2.6% of cases. In previous studies, cognitive impairment was documented in up to 42% of cases [30,31].

PD clinical phenotypes can also determine PD patients’ quality of life. PIGD phenotype PD patients had a higher frequency of non-motor symptoms [32]. Non-motor symptoms have been described as more severe in PIGD-type PD compared with tremor dominant PD, with sleep impairment and fatigue being the most important factors affecting PD patients’ QoL [33]. In our study, we obtained statistically significant differences between PD subtypes with respect to constipation problems, daytime sleepiness, lightheadedness on standing, and depressed mood, indicating that PIGD is more severe. A comparison of PD subgroups showed that the PIGD group had a higher prevalence of all non-motor symptoms, whereas the prevalence of non-motor symptoms in the TD group was consistent with the results of the other surveys, except for fatigue. In our study, fatigue had a higher prevalence in both PD subgroups compared to previous studies.

According to the SCOPA-AUT questionnaire results, patients with the PIGD phenotype had severe manifestations of non-motor symptoms in all domains and in total scores. Among these, gastrointestinal and urinary function were the only symptoms with statistically significant differences between the groups; the PIGD subtype had worse scores than the TD subgroup did. According to the literature, worse SCOPA-AUT scores have been indicated in the PIGD subtype. Interestingly, there were no statistically significant differences between the two subgroups’ SCOPA-AUT pupillomotor and sexual scores [34], as was also the case in our study. It is probable that had our study included a larger population, we could have obtained more statistically significant differences between the PD patient subgroups regarding all parts of the SCOPA-AUT questionnaire. Only 20 PD patients completed the SCOPA-AUT sexual domain questionnaire, possibly for ethical reasons.

Analysis of results showed differences in the PD subgroups’ motor assessments. PIGD patients had higher motor scores according to all parts of the MDS-UPDRS and HY stages. Previous studies have shown that PIGD-type PD is associated with more severe motor and cognitive impairment, a more rapidly progressive course, and a higher risk of disability. This PD phenotype has a steeper slope than the tremor dominant group in all UPDRS parts I–III and in total, with a worse prognosis [35,36]. Additionally, our research presents a correlation with the MDS-UPDRS total score and scores for parts II, III and IV being worse in the PD PIGD subtype.

The severity of disease symptoms affects patients’ quality of life. Among non-motor symptoms assessed using MDS-UPDRS part I, a moderate positive correlation was found for apathy, sleep problems and daytime sleepiness, pain and other sensations, lightheadedness on standing, and fatigue. The association between depression and Parkinson’s disease has been described as one of the most significant factors influencing PD patients’ QoL [37]; however, in our research, depressed and anxious mood had low positive correlations with the PDQ-39. Hallucinations and psychosis and urinary problems did not have statistically significant correlations with the PDQ-39. Notably, the total score, which included all non-motor symptoms, had high positive correlations with the PDQ-39. 

Patients’ complaints of cognitive impairment (according to the MDS-UPDRS scale part I) showed a moderate correlation with the PDQ-39 index. The MoCA test results regarding memory assessment also showed a low statistically significant correlation with the PDQ-39.

The symptom of lightheadedness on standing had a moderate positive correlation with the PDQ-39; when it was included in the SCOPA-AUT cardiovascular domain, the moderate positive correlation remained. A low positive correlation was obtained between constipation problems and the PDQ-39; when constipation problems were included in the SCOPA-AUT gastrointestinal domain together with other symptoms, the correlation was moderately positive. Urinary problems alone did not correlate with the PDQ-39; no correlation was found when calculated in the SCOPA-AUT urinary domain. Although our study did not find differences in the SCOPA-AUT thermoregulatory and pupillomotor domains between PD subgroups, both of these domains had moderate correlations with patients’ quality of life.

In one of the most extensive Japanese studies (2020), which included more than 3000 patients, all non-motor symptoms were reported as being prevalent and negatively affecting patients’ quality of life [38]. Our study showed that fatigue was the most common non-motor symptom and that it had a moderate correlation with the PDQ-39. Therefore, understanding how non-motor symptoms affect patients’ quality of life and recognizing these symptoms is important. A previous study demonstrated that neurologists failed to identify depression, anxiety, and fatigue symptoms more than half of the time [39].

The strength of this study was its comprehensive assessment of disease symptoms, including a broad spectrum of non-motor and motor symptoms, and disease severity by one trained neurologist, which reduced the potential for bias. However, our study had limitations. First, this was a single-center study with a small group of PD patients. Secondly, although the MDS-UPDRS captures a wide range of PD manifestations, and is therefore useful when studying the relationship between PD-related impairments and quality of life, additional scales with more detailed questions should be used to assess PD symptoms such as fatigue, sleep problems, depression, and anxiety in greater depth.

## 5. Conclusions

We present our study as the first conducted in Latvia that analyzed the prevalence of non-motor and motor symptoms and the impact of PD symptoms on health-related quality of life. Our research shows that motor and non-motor symptoms of Parkinson’s disease significantly affect patients’ quality of life. As the disease progresses and the degree of disability increases, patients’ quality of life and self-care abilities deteriorate.

Our research shows that, similar to motor symptom severity and the Hoehn and Yahr stage, PD patients’ non-motor symptoms, such as fatigue, also negatively affect their health-related quality of life. It is also important to remember the influence of the thermoregulatory and pupillomotor function domains, which, according to our data, significantly affect the well-being of patients along with other disease symptoms.

Patients with the PIGD phenotype had a severe presentation of motor and non-motor symptoms compared to tremor dominant PD, except for fatigue. Further studies with a larger number of patients and more centers for the evaluation of Parkinson’s disease symptoms in the Latvian population are needed.

## Figures and Tables

**Table 1:** Frequency of symptoms according to MDS-UPDRS part I among Parkinson’s disease clinical phenotypes.

	All	Parkinson’s Disease Clinical Phenotypes		
MDS-UPDRS Part I		TD	PIGD	*p*-Value
	*n* (%)	*n* (%)	*n* (%)	
MDS-UPDRS part I (1.1–1.6)	*n* = 42	*n* = 14	*n* = 24	
Cognitive impairment	34 (81%)	11 (78.6%)	20 (83.3%)	0.157
Hallucinations and psychosis	3 (7.1%)	0	3 (12.5%)	0.24
Depressed mood	27 (64.3%)	6 (42.9%)	19 (79.2%)	0.035
Anxious mood	18 (42.9%)	5 (35.7%)	10 (41.7%)	0.717
Apathy	24 (57.1%)	6 (42.9%)	15 (62.5%)	0.24
Features of DDS	6 (14.3%)	0	6 (25%)	0.067
MDS-UPDRS part I (1.7–1.13)	*n* = 43	*n* = 14	*n* = 25	
Sleep problems	36 (83.7%)	10 (71.4%)	23 (92%)	0.163
Daytime sleepiness	36 (83.7%)	9 (64.3%)	24 (96%)	0.016
Pain and other sensations	35 (81.4%)	9 (64.3%)	22 (88%)	0.109
Urinary problems	30 (69.8%)	8 (57.1%)	21 (84%)	0.124
Constipation problems	28 (65.1%)	5 (35.7%)	21 (84%)	0.004
Lightheadedness on standing	25 (58.1%)	4 (28.6%)	17 (68%)	0.018
Fatigue	41 (95.3%)	13 (92.9%)	24 (96%)	1

Abbreviations: MDS-UPDRS = Movement Disorder Society-sponsored revision of the Unified Parkinson’s Disease Rating Scale; DDS = dopamine dysregulation syndrome.

**Table 2:** Non-motor (SCOPA-AUT) and motor (MDS-UPDRS) scores of PD patients with TD and PIDG phenotypes.

Domain	Total		Parkinson’s Disease Clinical Phenotypes				*p*-Value
			TD		PIGD		
	Median	Q1:Q3	Median	Q1:Q3	Median	Q1:Q3	
SCOPA-AUT							
Gastrointestinal function	3.00	1.00–5.00	1.00	0–3.00	4.00	2.00–6.00	0.001
Urinary function	4.50	3.00–7.00	3.00	1.50–5.00	6.00	3.50–8.50	0.01
Cardiovascular function	1.00	0–2.00	0	0–1.50	1.00	0–2.00	0.097
Thermoregulatory function	2.00	0.75–5.00	1.00	0–3.00	3.00	1.00–5.50	0.06
Pupillomotor function	0	0–1.00	0	0–0.50	1.00	0–1.00	0.06
Sexual function	1.00	0–2.75	2.00	0–3.00	0.50	0–2.00	0.34
Total score	12.00	7.75–18.50	8.00	4.50–12.50	13.00	10.50–24.00	0.003
MDS-UPDRS							
Part I	11.00	8.00–19.00	7.00	5.00–11.25	17.00	11.00–21.50	<0.001
Part II	13.00	6.00–21.00	6.50	3.75–11.00	19.00	13.00–23.00	<0.001
Parts III and IV	35.00	27.00–41.00	29.50	22.25–38.25	39.00	32.50–51.00	0.008
Total score	61.00	42.00–80.00	48.50	33.25–59.50	75.00	57.00–88.50	<0.001
HY scale	2	1–3	1	1–2	2	1.5–3	0.01

Abbreviations: SCOPA-AUT = scale for outcomes in Parkinson’s disease for autonomic symptoms; MDS-UPDRS = Movement Disorder Society-sponsored revision of the Unified Parkinson’s Disease Rating Scale; HY scale = Hoehn and Yahr scale.

**Table 3:** Correlations of health-related quality of life (assessed using the PDQ-39 index) with non-motor and motor scores of Parkinson’s disease patients.

Scales/Subscales	Spearman’s Rank Correlation Coefficient with the PDQ-39 Index	*p*-Value
Disease duration (y)	0.529	<0.001
SCOPA-AUT total score	0.654	<0.001
Gastrointestinal function	0.639	<0.001
Urinary function	0.25	0.11
Cardiovascular function	0.586	<0.001
Thermoregulatory function	0.566	<0.001
Pupillomotor function	0.597	<0.001
Sexual function	0.132	0.579
MDS-UPDRS part I	0.778	<0.001
Cognitive impairment	0.571	<0.001
Hallucinations and psychosis	0.182	0.254
Depressed mood	0.362	0.02
Anxious mood	0.331	0.034
Apathy	0.6	<0.001
Features of DDS	NA	NA
Sleep problems	0.581	<0.001
Daytime sleepiness	0.596	<0.001
Pain and other sensations	0.64	<0.001
Urinary problems	0.243	0.121
Constipation problems	0.407	0.008
Lightheadedness on standing	0.663	<0.001
Fatigue	0.583	<0.001
MDS-UPDRS part II	0.784	<0.001
MDS-UPDRS parts III and IV	0.704	<0.001
MDS-UPDRS total score	0.837	<0.001
HY scale	0.723	<0.001
MoCA test	0.438	0.006

Abbreviations: SCOPA-AUT = scale for outcomes in Parkinson’s disease for autonomic symptoms; MDS-UPDRS = Movement Disorder Society-sponsored revision of the Unified Parkinson’s Disease Rating Scale; HY scale = Hoehn and Yahr scale; MoCA test = Montreal cognitive assessment test; PDQ-39 = 39-item Parkinson’s Disease Quality of Life Questionnaire; DDS = dopamine dysregulation syndrome; NA = not applicable; y = year.

## Data Availability

Data presented in this study are available on request from the corresponding author. Data are not publicly available due to ethical restrictions.
